# Lateral growth of cylinders

**DOI:** 10.1038/s41467-022-29863-8

**Published:** 2022-04-21

**Authors:** Hui Sun, Shuai Chen, Xiao Li, Ying Leng, Xiaoyan Zhou, Jianzhong Du

**Affiliations:** 1 State Key Laboratory of High-Efficiency Coal Utilization and Green Chemical Engineering, Ningxia University, 750021 Yinchuan, China; 2 Department of Gynaecology and Obstetrics, Shanghai Fourth People’s Hospital, School of Medicine, Tongji University, 200434 Shanghai, China; 3 Department of Polymeric Materials, School of Materials Science and Engineering, Tongji University, 4800 Caoan Road, 201804 Shanghai, China

**Keywords:** Supramolecular polymers, Self-assembly, Self-assembly, Polymers, Polymers

## Abstract

The precise control of the shape, size and microstructure of nanomaterials is of high interest in chemistry and material sciences. However, living lateral growth of cylinders is still very challenging. Herein, we propose a crystallization-driven fusion-induced particle assembly (CD-FIPA) strategy to prepare cylinders with growing diameters by the controlled fusion of spherical micelles self-assembled from an amphiphilic homopolymer. The spherical micelles are heated upon glass transition temperature (*T*_g_) to break the metastable state to induce the aggregation and fusion of the amorphous micelles to form crystalline cylinders. With the addition of extra spherical micelles, these micelles can attach onto and fuse with the cylinders, showing the living character of the lateral growth of cylinders. Computer simulations and mathematical calculations are preformed to reveal the total energy changes of the nanostructures during the self-assembly and CD-FIPA process. Overall, we demonstrated a CD-FIPA concept for preparing cylinders with growing diameters.

## Abstract (web summary)

Precise control of the microstructure of nanomaterials is of interest but living lateral growth of cylinders is still very challenging. Here, the authors propose a crystallization-driven fusion-induced particle assembly strategy to prepare cylinders with growing diameters by controlled fusion of spherical micelles self-assembled from an amphiphilic homopolymer.

## Introduction

Macromolecular self-assembly is a powerful tool to fabricate functional soft nanomaterials with diverse morphologies including spherical micelles, cylindrical micelles, vesicles and nanobowls, etc.^1–6^. Among them, cylindrical micelles, also known as cylinders, are appealing and versatile due to their unique serpentine conformation and adjustable aspect ratio comparing with their spherical counterparts. These cylinders have shown considerable potentials in cellular uptake and in vivo drug delivery due to their high aspect ratio^7–10^. The control of geometric size such as the length and diameter of cylinders is very important and has drawn much attention in the last decade^11–14^. Traditional self-assembly of amphiphilic polymers can regulate the length and diameter of cylinders by changing the chemical composition of polymers, such as the length of hydrophobic block. Taking advantage of the “living” characteristic, the crystallization-driven self-assembly (CDSA) strategy has an aptitude for the control of the length of cylinders^15–17^. Upon addition of polymers, the crystalline or semicrystalline segments crystallized along with the core and guarantee the living longitudinal growth of cylinders^18–20^. For instance, Manners and coworkers developed seeded growth of cylindrical micelles based on the highly crystalline poly(ferrocenyldimethylsilane) (PFS)^21^, and the cylindrical micelles could grow on different manners, such as from both ends^22^, from one end^23^ and using different blocks^24^. O’Reilly et al. prepared biodegradable cylinders with controlled length from the semicrystalline polymers including poly(ʟ-lactide) (PLLA)^25^, poly(*ε*-caprolactone) (PCL)^26,27^ based block copolymers via CDSA benefiting from the crystalline property of PLLA and PCL. Recently, Lin and coworkers studied the controlled growth and termination of cylinders self-assembled from liquid-crystallization-driven self-assembly of poly(*γ*-benzyl-ʟ-glutamate)-*block*-poly(*N*-isopropyl acrylamide) (PBLG-*b*-PNIPAM) from both of theoretical and experimental aspect^28^. However, the lateral growth of cylinders is still challenging. Despite the changing of the length of the hydrophobic or the crystalline blocks to control the diameter of cylinders, the living lateral growth of cylinders is still hard to achieve.

The fusion and assembly of particles is an emerging method to fabricate nanomaterials with precisely controlled anisotropy or highly ordered structures^29–33^. For instance, Yuan and coworkers fabricated AB_*n*_-type colloidal particles by polymerization-induced particle-assembly using a linear ABC triblock terpolymer, and the number of *n* could be controlled between 2 and 6 by tuning the volume ratio of different blocks^29^. O’Reilly et al. investigated the polymerization-induced fusion behaviors of polymersomes^33^. They found that the membrane tension of the polymer vesicles with the continued polymerization was the main driving force for the fusion of polymersomes to form tubesomes. Recently, our group proposed a FIPA strategy to prepare tetrapod polymersomes and micelle clusters by spherical polymersomes and micelles^30^. The formation of either tetrapod polymersomes or micelle clusters is a matter of balance between pro-fusion and anti-fusion forces provided by the different components in the polymer. One step further, giant polymer vesicles with a latticelike membrane were also obtained by the FIPA of small-sized vesicles^34^.

**Fig. 1:**
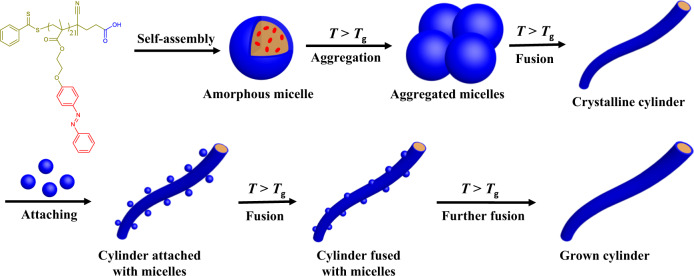
Self-assembly of PAzoMA to form spherical micelles and living lateral growth of cylinders by CD-FIPA of micelles. PAzoMA homopolymer can self-assemble into amorphous spherical micelles, which aggregate and fuse to form crystalline cylinders when incubated at 75 °C. With the addition of spherical micelles, the micelles attach onto and fuse with the cylinders, leading to the living lateral growth of cylinders.

In this work, we propose a CD-FIPA strategy to achieve the living lateral growth of cylinders via the hierarchical self-assembly of a semicrystalline homopolymer, as shown in Fig. 1. The amphiphilic homopolymer poly(2-(4-(phenyldiazenyl)phenoxy)ethyl methacrylate) (PAzoMA) with only hydrophilic terminal carboxylic group and crystalline azobenzene pendents was synthesized via reversible addition-fragmentation chain transfer (RAFT) polymerization. Upon addition of water to the solution of PAzoMA, spherical micelles were obtained. However, the colloidal stability of the micelles is broken when the temperature rises to 75 °C (above the *T*_g_ of PAzoMA), leading to the aggregation and fusion of the micelles, as well as the formation of cylinders. Interestingly, the amorphous to crystalline transformation occurred between spherical micelles and cylinders due to the regular rearrangement of the azobenzene pendants since the incubation temperature is higher than the *T*_g_ (63.1 °C) of PAzoMA to ensure the sufficient motion of azobenzene pendants. More importantly, the diameter of the cylinders can be controlled from 68.6 ± 12.3 to 137.6 ± 17.3 nm with the addition of spherical micelles to the solution of cylinders. In addition, the cross-section area of the cylinders shows a linear relationship with the mass ratio of added spherical micelles to cylinders, indicating that all the spherical micelles added are fused with the cylinders. Moreover, computer simulation and theoretical calculation further confirm that the CD-FIPA of spherical micelles to form cylinders is a process of reducing the energy of the system, resulting in a more stable thermodynamic state.

## Results and discussion

### Synthesis, characterization, and self-assembly of PAzoMA

The PAzoMA was synthesized via RAFT polymerization using 4-cyano-4-((phenylcarbonothioyl)thio)pentanoic acid (CPAD) as chain transfer agent. The synthetic route of AzoMA monomer and PAzoMA was presented in Supplementary Fig. 1. The nuclear magnetic resonance (NMR) and high resolution mass spectra in Supplementary Figs. 2 and 3a–c demonstrated the successful synthesis of the AzoMA monomer. The disappearance of the signal of the double bond in the NMR spectrum in Supplementary Fig. 4a indicated the entire consumption of AzoMA monomer, and the ^13^C NMR spectra in Supplementary Fig. 4b further confirmed the successful synthesis of PAzoMA. The degree of polymerization of PAzoMA was 21, as calculated from the conversion of the monomer based on the NMR spectrum in Supplementary Fig. 4a. The number average molecular weight of PAzoMA was calculated to be 6900. The gel permeation chromatography (GPC) trace of PAzoMA in Supplementary Fig. 5 revealed a molecular weight of 6700 with a low polydispersity index (*Đ*) of 1.11, which was in accordance with the molecular weight calculated from the NMR spectrum.

The thermal property of PAzoMA was investigated by differential scanning calorimetry (DSC), as illustrated in Supplementary Fig. 6. The *T*_g_ of PAzoMA was 63.1 °C, while the melting point was 100.4 °C. The melting enthalpy (Δ*H*_m_) of PAzoMA was measured to be 6.62 J g^‒1^, which is much lower than that of reported typical crystalline and azobenzene-containing polymers^35,36^. Assuming that a purely crystalline sample has a typical value of Δ*H*_m_ ≈ 150−300 J g^−1,^^37^ the crystallinity of PAzoMA is estimated to be ~ 4−8%. The crystal structure of PAzoMA at different temperatures (25, 50, 75, 100 and 110 °C) was characterized by powder X-ray diffraction (XRD), as shown in Supplementary Fig. 7. There are three weak diffraction peaks centered at 2*θ* of 31.6°, 36.5°, and 45.1°, corresponding to the (120), (114), and (700) plane of PAzoMA^38–40^. When the temperature is elevated above the phase transition temperature of PAzoMA, the diffraction peaks in Supplementary Fig. 7 vanished, demonstrating the disappearance of the ordered structure. In addition, no liquid crystalline phase was observed in polarizing optical microscope (POM) images at 110 °C or cooling to 25 °C, as illustrated in Supplementary Fig. 8a, b, which might be ascribed to the lack of flexible or polar groups at the para position of azobenzene.

The PAzoMA has an ultralow hydrophilic content of 0.65% as its backbone and pendants are hydrophobic, while only the terminal carboxyl group is hydrophilic. Previous studies showed that very small amount of terminal groups could induce the self-assembly of amphiphilic homopolymers^41,42^. The PAzoMA could self-assemble into spherical micelles with a hydrodynamic diameter (*D*_h_) of 33 nm and low polydispersity (PD) of 0.107 at a water dropping rate of 0.5 mL min^‒1^, as measured by dynamic light scattering (DLS, Supplementary Fig. 9). In addition, the Zeta potential of the micelles was measured to be −23.9 mV (Supplementary Fig. 10), demonstrating that the terminal carboxyl groups covered the surface of the micelles, which kept the electrosteric stability of the micelles. The TEM image in Supplementary Fig. 11 revealed the spherical morphology of the micelles. The formation of the spherical micelles is kinetically controlled. When decreasing the water dropping rate to 0.1 mL min^‒1^, large compound micelles were obtained (Supplementary Fig. 12). After the formation of micelles, the UV–vis adsorption peak of PAzoMA was red shifted from 343 to 385 nm (Supplementary Fig. 13), indicating the strong *π–π* interaction between azobenzene pendants^43^.

### CD-FIPA of amorphous micelles to form crystalline cylinders

**Fig. 2:**
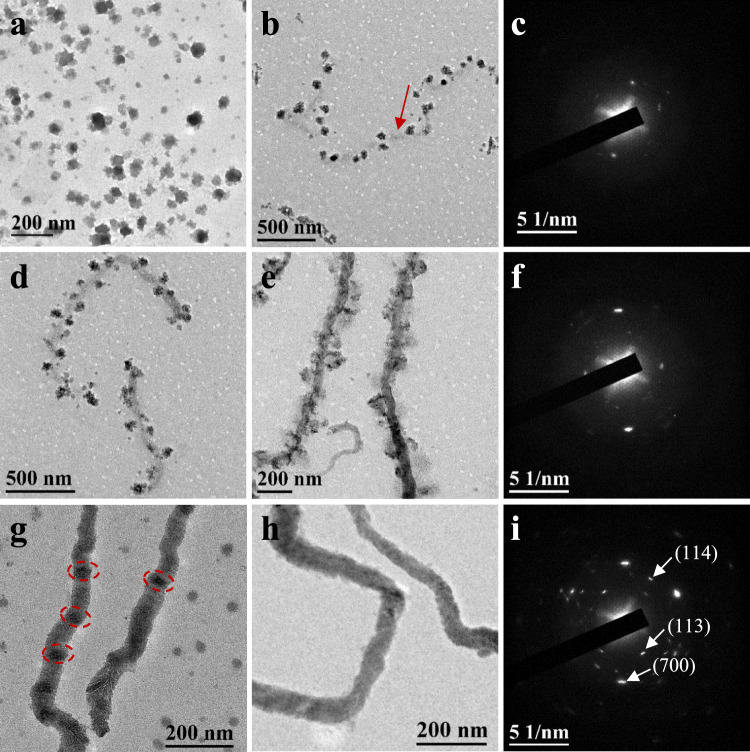
Formation of semicrystalline cylinders via CD-FIPA of spherical micelles. **a** Incubated for 15 min, **b** 30 min, and **c** corresponding SAED pattern; **d** 60 min, **e** 90 min, and **f** corresponding SAED pattern; **g** 3 h, **h** 6 h, and **i** corresponding SAED pattern.

Considering that the electrosteric stability of the micelles was mainly provided by the negative charge on the surface, we speculated that the thermo-treatment might disturb the stability of the micelles, which was a commonly used method to break the stability of colloids^44^. So the spherical micelle solution with a concentration of 0.12 mg mL^‒1^ was incubated in water bath at 75 °C to induce the aggregation and fusion of the micelles. As shown in Fig. 2a, the small spherical micelles aggregated and fused to form large particles with irregular morphology when incubated for 15 min. The DLS and Zeta potential results in Supplementary Figs. 9 and 10 further confirmed the aggregation of the micelles since the diameter increased to 68 nm. As the incubation time increased to 30 min, the irregular aggregates connected into a necklace to decrease the surface free energy and part of the polymers softened and connected to form the continuous structure, as pointed by the red arrow in Fig. 2b. Surprisingly, the connected micelles was semicrystalline rather than the amorphous state of the original spherical micelles (Supplementary Fig. 14), as shown in the selected area electron diffraction (SAED) pattern in Fig. 2c, demonstrating the regular rearrangement of the azobenzene pendants when incubated at 75 °C.

With the increase of incubation time, the connected micelles were fused to form cylindrical structures attached with fragments of spherical micelles, as presented in Fig. 2d, e. Besides, the electron diffraction signal in Fig. 2f demonstrated the preservation of the crystal structure during incubation. When the incubation time was extended to 3 h, the aggregates attached onto the cylindrical structures disappeared and the cylinders were formed by the CD-FIPA of spherical micelles. As shown in Fig. 2g, the high contrast areas pointed by the red circles were the cores of the spherical micelles that were not completely fused. In addition, there were still unfused spherical micelles in the solution. With the further prolongation of incubation time to 6 h, the ‘mature’ cylinders were formed (Fig. 2h), and the DLS and Zeta potential measurements revealed a *D*_h_ of 327 nm and a similar Zeta potential of −22.6 mV to the spherical micelles (Supplementary Figs. 9 and 10).

To investigate the influence of the concentration of spherical micelles on the particle fusion rate, the solution of spherical micelles was concentrated to 0.25 mg mL^−1^ and incubated at 75 °C. As shown in Supplementary Fig. 15a–c, the particle fusion rate barely changed, which might be controlled by the crystallization process of the homopolymer. Supplementary TEM image of the preformed cylinders at low magnification was provided in Supplementary Fig. 16, revealing a diameter of 56.2‒80.4 nm (average diameter of 68.6 ± 12.3 nm) and a length of 1110‒1940 nm. The cylinder has gradient diameter with thick head and thin tail, which is formed at the very beginning of the formation of cylinders, as illustrated in Supplementary Fig. 17. The morphology of the cylinders was similar to the previously reported cylinders that obtained via counterion-mediated particle fusion of spherical micelles self-assembled from ion-containing block copolymers^45^. In addition, the diffraction spots in SAED pattern in Fig. 2i belong to the (113), (114), (700) planes of PAzoMA, corresponding to *d*-spacing of 0.331, 0.234 and 0.202 nm, respectively, which are consistent with the XRD results.

**Fig. 3:**
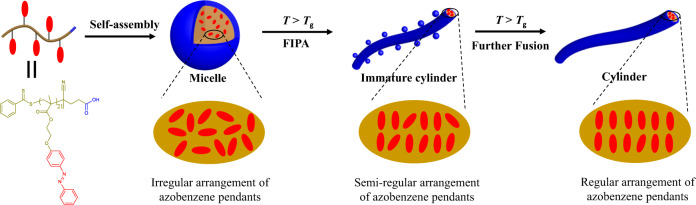
Proposed mechanism of the transformation from amorphous micelles to crystalline cylinders during CD-FIPA. The azobenzene pendants arrange randomly in the amorphous micelles. When incubated at 75 °C (higher than the *T*_g_ of PAzoMA), the chain mobility of PAzoMA significantly increases, leading to the regular arrangement of azobenzene pendants and crystalline structure of cylinders.

The proposed mechanism of the transformation from amorphous to crystalline during CD-FIPA is illustrated in Fig. 3. When the PAzoMA self-assembles into micelles, the polymer chains and the azobenzene pendants are arranged randomly, leading to the amorphous state of the micelles. As the temperature rises above the *T*_g_ of the PAzoMA, the micelles are fused to form necklace-like structure (Fig. 2b, e) and immature cylinders due to the disturbance of the electrosteric stability of the micelle solution, while the chain mobility of the PAzoMA significantly increases to promote the rearrangement of azobenzene pendants at the same time. Therefore, the obtained cylinders are crystalline instead of the amorphous state of the spherical micelles. With the prolonged incubation of the immature cylinders at 75 °C (>*T*_g_), the micelles attached onto the cylinder are entirely fused with the cylinders to form mature cylinders. The rearrangement of the azobenzene pendants and the CD-FIPA process should occur simultaneously.

### Living lateral growth of cylinders

**Fig. 4:**
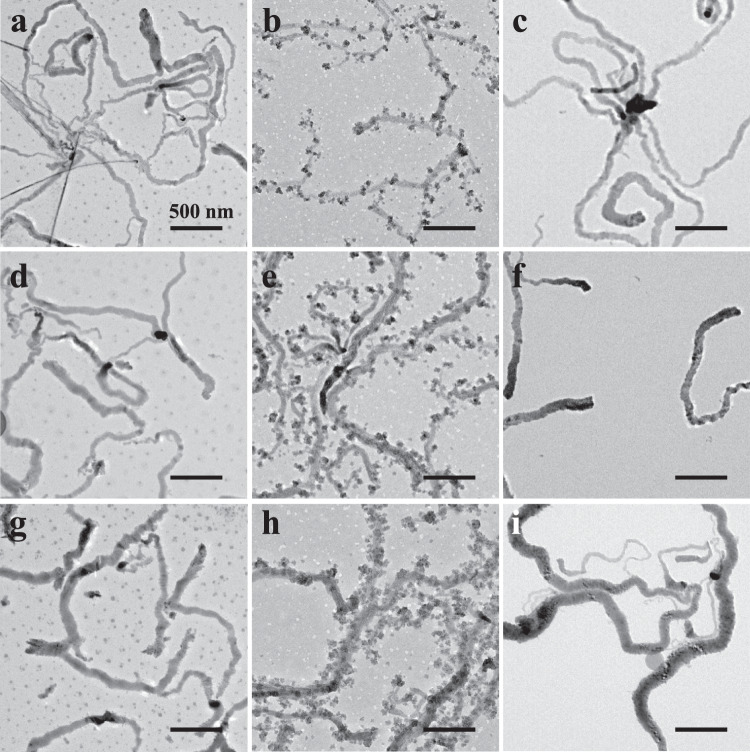
The living lateral growth of cylinders by CD-FIPA. **a**, **d**, and **g** the mixture of spherical micelles and cylinders before incubation; **b**, **e**, and **h** incubation at 75 °C for 60 min; **c**, **f**, and **i** incubation at 75 °C for 3 h.

Living lateral growth of cylinders is still challenging despite the changing of the length of hydrophobic chains to control the diameter of cylinders. Herein, we intended to conquer this challenge by the CD-FIPA of spherical micelles. As shown in Fig. 4a, the cylinders and spherical micelles were mixed and incubated at 75 °C. After 60 min, the micelles aggregated to form irregular aggregates and attached onto the surface of preformed cylinders (Fig. 4b). The darkness of the aggregates is intensive after attaching onto the cylinders, which is ascribed to the crystallization of the aggregates during the incubation process, as shown in Supplementary Fig. 18a, b. When the incubation time was prolonged to 3 h, the irregular aggregates were entirely fused with the preformed cylinders (Fig. 4c). In the previous section, we have proven that the formation of cylinders is due to the crystallization-driven aggregation and fusion of spherical micelles, which is also responsible for the lateral growth of cylinders. To further prove this hypothesis, the mixed solution of spherical micelles and cylinders was kept at 25 °C for 3 h. As illustrated in Supplementary Fig. 19, the added spherical micelles did not attach on the cylinders. In addition, the lateral grown sections of cylinders are also crystalline, as confirmed by the SAED patter in Supplementary Fig. 18b.

In order to achieve the living lateral growth of the cylinders, we repeated the above micellar addition procedure for two more times. As shown in Fig. 4d–f, the spherical micelles aggregated, attached and fused with the cylinders in a similar behavior. Upon addition of the spherical micelle solution for the third time, the diameter of the cylinders further increased (Fig. 4g–i), demonstrating the validity of the living lateral growth of the cylinders via the CD-FIPA of spherical micelles. The diameter of the cylinders increased to 94.1 ± 12.8 nm, and further increased to 118.3 ± 15.6 and 137.6 ± 17.3 nm after growth, respectively. More TEM images at low and high resolutions were provided in Supplementary Fig. 20a–f. The epitaxial growth of the cylinders was not observed from TEM images since the added spherical micelles attached onto the surface of the preformed cylinders instead of from the termini of the cylinders. Besides, the length of the cylinders was measured to be 1200–1920 nm, which was consistent with that before growth, further excluding the termini growth of the cylinders. Furthermore, the laterally grown cylinders inherited the gradient diameter features of the preformed cylinders, indicated that the added micelles attached onto and fused with the cylinders evenly, further proving the validity of the lateral growth of cylinders. There are few thin cylinders in the lateral growth process of cylinders, as illustrated in Fig. 4d, i, which is ascribed to the entire consumption of added spherical micelles, so there are no micelles attached onto the surface of the cylinders, as pointed by the red arrows in Supplementary Fig. 21. It is noteworthy that thin cylinders were reduced when the mass ratio of added spherical micelles to cylinders was increased to two times of that before. As presented in Supplementary Fig. 22a, b, the diameter of the cylinders is more uniform than before and thin cylinders disappeared, demonstrating the controllability of the living lateral growth of cylinders by CD-FIPA.

**Fig. 5:**
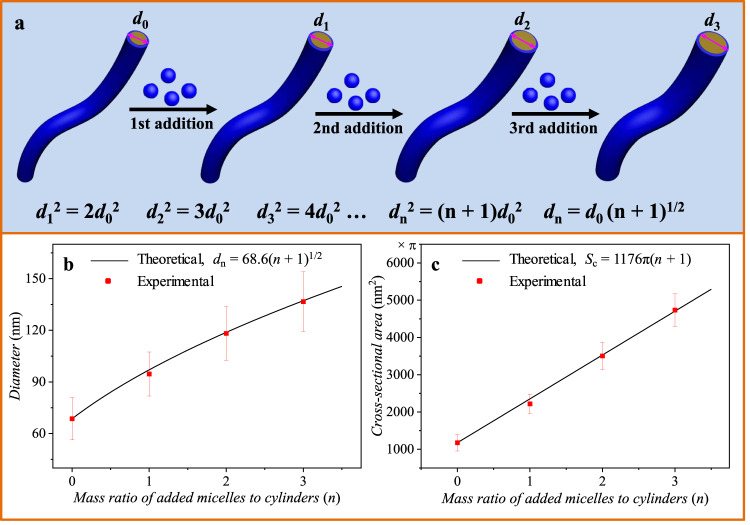
Quantitative analysis of the living lateral growth of the cylinders. **a** Schematic illustration of the living lateral growth of cylinders, and the relationship between **b** diameter and **c** cross-sectional area of the cylinders and the mass ratio of added micelles to cylinders. The error bars in **b** and **c** are calculated by measuring the diameter of cylinders.

To reveal the living growing rule of the CD-FIPA of the spherical micelles on the surface of cylinders, we theoretically analyzed the relationship between the diameters (*d*) of the cylinders and the mass ratio of added micelles to cylinders (defined as *n*, *n* = 0 representing the preformed cylinders). In principle, the square of the diameter of grown cylinders should have a linear relationship with (*n* + 1), as shown in Fig. 5a. The deduction details are presented in Supplementary information. In reality, the experimental results confirmed this mathematical prediction. As illustrated in Fig. 5b, theoretically, the diameter of the grown cylinders shows a square root relationship with (*n* + 1). Indeed, the experimental results were well fitted with the theoretical prediction. Similarly, the cross-sectional area (*S*_c_) of the cylinders showed a linear relationship with (*n* + 1), as presented in Fig. 5c. In the previous section, we have confirmed that the length of the cylinders remains the same before and after growth. Assuming that the density of the spherical micelles and cylinders remained unchanged before and after growth of cylinders, the total volume of the cylinders should have a linear relationship with (*n* + 1), demonstrating that all the spherical micelles were attached onto and fused with the cylinders, which could also be confirmed by the TEM images in Fig. 4c, f and i.

### Computer simulations to reveal the energy fluctuation during self-assembly and CD-FIPA

The conformation optimization of the PAzoMA and the energy evolution of the system during the self-assembly and CD-FIPA processes were monitored by molecular dynamics (MD) simulation by Materials Studio 2017 according to our previous study^46^. MD simulation is a thermodynamic calculation method which adopted Newton’s equations of motion for the calculation of all-atom trajectories, thus acquiring the thermodynamic quantities and other macroscopic properties of different molecular systems^47^. Typically, the structural changes and the energy evolution reflect the thermodynamic state of the system; the decreasing of the total energy of the “cell” indicates a more stable thermodynamic state under simulated forces. The details of the simulation settings were presented in the Supplementary information. First, we investigated the self-assembly of PAzoMA_21_ to form spherical micelles by MD simulation. As shown in Supplementary Fig. 23, the model of PAzoMA_21_ was built and optimized by Forcite, and the initial state and the output state of the “cell” containing 5 model chains were also presented in Supplementary Fig. 24a, b, revealing the optimization of the geometry of the molecular chains to decrease the energy of the system. In addition, the change of the total energy (Δ*E*) of the “cell” was calculated to be ‒12,953.2 kcal mol^‒1^ (Supplementary Fig. 25), demonstrating the decrease of the total energy during self-assembly to reach a more stable thermodynamic state.

**Fig. 6:**
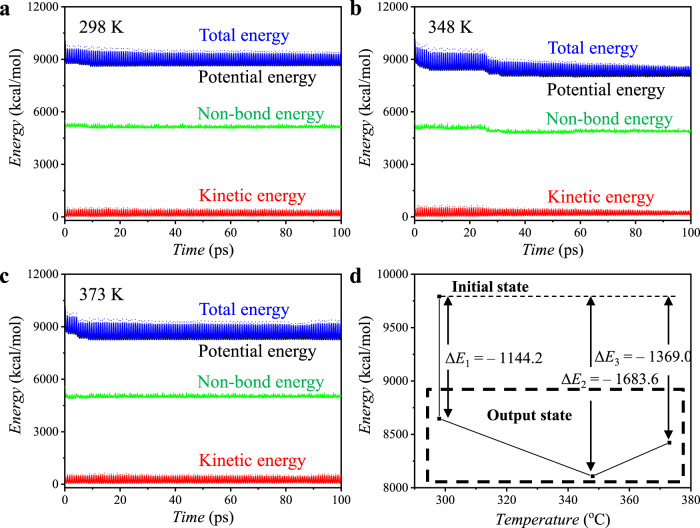
Energy evolution curves of PAzoMA_5_ in a crystalline cell from MD simulations at different temperatures. **a** 298, **b** 348, and **c** 373 K. **d** Illustrative analysis of energy state and Δ*E* calculated from MD simulations at 298, 348 and 373 K, respectively.

To investigate the geometry optimization of PAzoMA and the energy evolution during CD-FIPA process, MD simulation was further conducted by constructing a crystalline cell consisting of one PAzoMA_5_ chain to simplify the calculation process. The Forcite-optimized model of PAzoMA_5_ was illustrated in Supplementary Fig. 26, and the corresponding initial state and output state of the molecular geometry at different temperatures were also investigated and presented in Supplementary Fig. 27a–d. As shown in Fig. 6, the energy evolution of the “cell” and the summary of the energy state, as well as the change of Δ*E* at different temperatures were also obtained. The total energy of the “cell” is 9791.9 kcal mol^‒1^, which descended to different levels at 298, 348, and 373 K (Fig. 6a–c). However, the Δ*E* reached a lowest state at 348 K, which is slightly higher than the *T*_g_ of the PAzoMA, corresponding to a Δ*E* of ‒1683.6 kcal mol^‒1^, as illustrated in Fig. 6d. The lowest energy state of the “cell” at 348 K demonstrated that the system reached the most stable thermodynamic state, so we conducted CD-FIPA at this temperature.

### Mechanism of CD-FIPA

We have confirmed that all the spherical micelles were fused to form cylinders. Considering that the specific surface area of the spherical micelles was significantly decreased when aggregating to cylinders, we quantitatively calculated the change of the specific surface area of the spherical micelles before and after the formation of cylinders. The relationship between the specific surface area of the cylinders and the spherical micelles was illustrated in Eq. (1). The detailed calculation process was presented in Supplementary information. The average radiuses of the spherical micelles and cylinders measured from TEM images are 12.4 and 34.3 nm, respectively. Therefore, the ratio of *S*_2_ to *S*_1_ equals to 0.241, which means that the total specific surface area of the spherical micelles decreased by 75.9% after CD-FIPA to form cylinders.

1 $$\frac{{S}_{2}}{{S}_{1}}{{\mbox{=}}}\frac{2{r}_{1}}{3{r}_{2}}$$

where *r*_1_ and *r*_2_ represent the average radiuses of the spherical micelles and the cylinders; *S*_1_ and *S*_2_ are the total specific surface areas of the spherical micelles and the cylinders.

In addition, we calculated the changes of the total specific surface area of the system before and after the lateral growth of cylinders. The following Eq. (2) was obtained and the detailed calculation process was also presented in the Supplementary information. The summary of the relative specific surface area of the cylinders after growth calculated from Eq. (2) was presented in Table 1. The mass ratio of added micelles to cylinders (*n*) of 0 means the CD-FIPA of the spherical micelles to form preformed cylinders, which has been discussed in the previous subsection. With the addition of spherical micelles to the solution of cylinders, the micelles attached onto and fused with the cylinders to form grown cylinders with increased diameters. The total specific surface area of the added micelles was decreased by 63.0% when fusing with the preformed cylinders for one time. Repeating this process, the living lateral growth of cylinders could be achieved. The relative specific surface areas of the grown cylinders were decreased by 62.4% and 54.5% comparing with the total specific surface area of the added micelles and premature cylinders, respectively, demonstrating the lower surface free energy of the grown cylinder.

2 $$\frac{{S}_{3}}{{S}_{1}{{\mbox{+}}}{S}_{2}}{{\mbox{=}}}\frac{2{r}_{1}{r}_{3}}{3\left({r}_{3}^{2}-{r}_{2}^{2}\right){{\mbox{+}}}{r}_{2}}$$

where *r*_1_ represents the average radiuses of the spherical micelles; *r*_2_ and *r*_3_ represent the average radiuses of the cylinders before and after growth; *S*_1_, *S*_2_, and *S*_3_ are the total specific surface area of the added spherical micelles and the cylinders before and after growth, respectively.

**Table 1:** Summary of the radiuses of the cylinders and relative specific surface areas at different mass ratio of added micelles to cylinders.

Mass ratio of added micelles to cylinders (*n*)	*r*_1_ (nm)	*r*_2_ (nm)	*r*_3_ (nm)	Relative *S*^a^
0	12.4	‒	‒	0.241
1	12.4	34.3	47.1	0.370
2	12.4	47.1	59.2	0.376
3	12.4	59.2	68.8	0.455

^a^Relative total specific surface area of the cylinders after growth comparing to that before aggregation and fusion with the spherical micelles, which was obtained by *S*_3_/(*S*_1_ + *S*_2_).

In summary, a CD-FIPA strategy was proposed to prepare crystalline cylinders from amorphous micelles and control the living lateral growth of cylinders. Upon increasing temperature, the electrosteric stability of the spherical PAzoMA micelles was broken, leading to the aggregation and fusion of the micelles to form cylinders. It is worth noting that the cylinders are crystalline, which is different from amorphous spherical micelles due to the regular rearrangement of the azobenzene pendants when incubated at 75 °C. More importantly, the living lateral growth of the cylinders could be accomplished by further attaching and fusion of the added spherical micelles with the cylinders. The diameter of the cylinders could be controlled from 68.6 ± 12.3 to 137.6 ± 17.3 nm. Either from experimental observation or theoretical calculation, we confirmed that crystallization is the main driving force of CD-FIPA, and all the added micelles were attached and fused with the cylinders. MD simulations confirmed that the total energy of the system decreased sharply during the self-assembly and CD-FIPA processes, demonstrating the more stable thermodynamic state of the cylinders. Besides, mathematical calculations also revealed that the total specific surface area of the cylinders were significantly decreased after CD-FIPA and lateral growth of the cylinders.

## Methods

### Reagents

Anhydrous ethanol (AR), 4-(phenyldiazenyl)phenol (98%), 2-bromoethanol (95%), K_2_CO_3_ (AR), methacryloyl chloride (95%), triethylamine (AR), tetrahydrofuran (THF, AR), dioxane (AR), NaHCO_3_ (AR), anhydrous Na_2_SO_4_ (AR), ethyl acetate (AR), DMF (HPLC), 4,4’-azobis(4-cyanovaleric acid) (98%) and 4-cyano-4-((phenylcarbonothioyl)thio)pentanoic acid (HPLC, > 97%) were purchased from Aladdin. CDCl_3_ was purchased from J&K Scientific, Ltd. THF was dried via sodium before use, and other reagents were used without further purification.

### Synthesis of 2-(4-(phenyldiazenyl)phenoxy)ethanol

4-(Phenyldiazenyl)phenol (3.962 g, 20.00 mmol) and 2-bromoethanol (3.099 g, 25.00 mmol) was dissolved in 100 mL of anhydrous ethanol in a round bottom flask. K_2_CO_3_ (3.455 g, 25.00 mmol) was added into the flask as acid binding agent. Then the dispersion was stirred at room temperature for 72 h. After reaction, the dispersion was filtered and dried under vacuum. The filter residue was dissolved in ethyl acetate and washed with saturated NaHCO_3_ and water for 3 times, respectively. The organic layer was dried via anhydrous Na_2_SO_4_ and dried under vacuum after filtration. The obtained crude product was recrystallized in ethanol to give purified product. ^1^H NMR (400 MHz, CDCl_3_, δ, ppm, Supplementary Fig. 2): 7.96–7.85 (m, 4H), 7.55–7.41 (m, 3H), 7.04 (d, *J* = 8.0 Hz, 2H), 4.16 (t, *J* = 4.0 Hz, 2H), 4.00 (t, J = 4.0 Hz, 2H).

### Synthesis of 2-(4-(phenyldiazenyl)phenoxy)ethyl methacrylate (AzoMA) monomer

The obtained 2-(4-(phenyldiazenyl)phenoxy) ethanol (2.421 g, 10.00 mmol) and triethylamine (1.518, 15.00 mmol) were dissolved in 30 mL of anhydrous tetrahydrofuran in a round bottom flask. The temperature was cooled to 0–5 °C. Methacryloyl chloride (1.375 g, 15.00 mmol) was diluted in 10 mL of anhydrous tetrahydrofuran and dropped into the flask in 20 min. Then the solution was stirred at room temperature for 24 h. After reaction, the dispersion was filtered and dried under vacuum. The obtained powder was dissolved in ethyl acetate and washed with saturated NaHCO_3_ and water for 3 times, respectively. The organic layer was dried via anhydrous Na_2_SO_4_ and dried under vacuum after filtration. The obtained crude product was purified by column chromatography (ethyl acetate/n-hexane = 1/3 (v/v)) to give AzoMA. ^1^H NMR (400 MHz, CDCl_3_, δ, ppm, Supplementary Fig. 3a): 7.96–7.85 (m, 4H), 7.55–7.41 (m, 3H), 7.04 (d, *J* = 8.0 Hz, 2H), 6.16 (s, 1H), 5.60 (s, 1H), 4.53 (t, *J* = 4.0 Hz, 2H), 4.29 (t, J = 4.8 Hz, 2H), 1.96 (s, 3H). ^13^C NMR (400 MHz, CDCl_3_, δ, ppm, Supplementary Fig. 3b): 167.30, 161.00, 152.74, 147.29, 135.95, 130.49, 129.06, 126.22, 124.78, 122.62, 114.89, 66.23, 62.93, 18.33. ESI-MS (*m*/z, Supplementary Fig. 3c): [M+H]^+^ calcd for C_18_H_18_N_2_O_3_; 311.1351; found, 311.1383.

### Synthesis of PAzoMA

The homopolymer PAzoMA was synthesized via RAFT polymerization according to the following procedures. CPAD (41.8 mg, 0.150 mmol), AzoMA (1.27 g, 3.75 mmol), and dioxane (2.0 mL) were added to a round bottom flask. Then oxygen was removed by filling with argon into the flask for 30 min. AIBN (3.70 mg, 0.0225 mmol) was rapidly added into the flask with bubbling for additional 5 min. Then the flask sealed with argon was placed in an oil bath at 70 °C. After 24 h, the reaction was terminated by cooling down to room temperature and exposing to air. The mixture was evaporated under vacuum to give a red-brown solid for further characterization. ^1^H NMR (400 MHz, CDCl_3_, δ, ppm, Supplementary Fig. 4a): 7.89–7.70 (1, m, 4H, (NC**CH**)_4_), 7.49–7.33 (a, b, n, o, 3H, (CH**CH**CH)_3_), 6.99–6.74 (c, k, 1H, OC**CH**CH), 4.40–3.83 (I, j, h, 4H, O**CH**_**2**_**CH**_**2**_O), 2.09–0.74 (d–g, 5H, C**CH**_**2**_C, C**CH**_**3**_). ^13^C NMR (400 MHz, CDCl_3_, δ, ppm, Supplementary Fig. 4b): 160.69, 152.60, 147.10, 130.49, 129.05, 124.77, 122.66, 114.68, 65.41, 63.18, 45.15, 44.80.

### Self-assembly of PAzoMA

The PAzoMA was dissolved in 5.0 mL of THF with a concentration of 0.5 mg mL^−1^, followed by adding deionized water (water/THF = 2:1 (v/v)) into the solution with a dropping rate of 0.5 mL min^‒1^ under stirring. The THF was removed by dialyzing against deionized water for 2 days to afford aqueous solution of spherical micelles (0.12 mL min^−1^).

### Formation of cylinders via the incubation of spherical micelles

The aqueous solution of spherical micelles with a concentration of 0.12 mg mL^‒1^ was incubated in a water bath at 75 °C. At different incubation time, part of the solution was taken out and cooled naturally for further characterization.

### Living lateral growth of cylinders

The aqueous solution of cylinders (0.12 mg mL^‒1^, 5 mL) was incubated in a water bath at 75 °C, then the aqueous solution of spherical micelles (0.12 mg mL^‒1^, 5 mL) was added into the solution and incubated for 3 h. After cooled to room temperature, the solution of cylinders with different diameters were obtained.

### Computer simulations

*Materials Studio* 2017 was used for MD simulations. The model for polymer was respectively packed into an amorphous cell or a crystalline cell via the module of Amorphous Cell. The module of Forcite was used to simulate the molecular structure adaptation and energy fluctuation during the self-assembly process of PAzoMA, and crystalline cell was used to reveal the energy fluctuation in the crystallization-driven fusion-induced particle assembly (CD-FIPA) of the micelles. Forcite module was used for geo-optimization of PAzoMA, MD simulations and energy calculation. Firstly, the corresponding models of the polymers used for simulations were constructed and optimized. PAzoMA_21_ was used for the calculation of the self-assembly process and PAzoMA_5_ without terminal group was used to reveal the CD-FIPA process in order to simplify the calculation procedure. All constructed models were optimized by the module of Forcite upon construction. The Forcite optimization parameters were as follows: algorithm: smart; convergence tolerance: energy: 1.0 × 10^−4^ kcal mol^−1^, force: 0.005 kcal mol^−1^ Å^−1^, displacement: 5.0 × 10^−5^ Å; maximum number of iterations: 500; motion groups rigid: NO; forcefield: universal. To test the structure optimization and energy fluctuation of the polymers during the self-assembly, different cells were built with the module of Amorphous Cell, respectively. For the self-assembly of PAzoMA_21_ to form micelles, five polymer chains were involved in one cell. While for the CD-FIPA process, one polymer chain was involved in the cell to simplify the calculation process. Afterward, the cell was calculated with dynamics under the parameters as follows: canonical (*NVT*); temperature: 298.00, 348.00, and 373.00 K; time step: 1.00 fs; number of steps: 50,000 or 100,000; and duration: 50 or 100 ps. The dynamics simulations were performed under universal forcefield. Energy evolution curves at different temperatures were obtained along with the simulation procedures. The energy data were obtained via the module of energy under Forcite to evaluate the corresponding energy changes of each cell quantitatively during the whole process of dynamics calculations.

## Supplementary information

Supplementary information

## Data Availability

All data needed to evaluate the conclusions of this study are available in the main text and Supplementary Information. The data supporting the findings of this work are available from the corresponding authors on request.
