# Supplementary material for: Lateral growth of cylinders
Source: Nat Commun. 2022 Apr 21;13:2170. doi: 10.1038/s41467-022-29863-8 (PMC9023456; doi:10.1038/s41467-022-29863-8)
Supplement: Supplementary file 1 — Supplementary information [file 41467_2022_29863_MOESM1_ESM.pdf]

Supplementary Information for

Lateral Growth of Cylinders

Hui Sun[1*], Shuai Chen[2,3], Xiao Li[1], Ying Leng[1], Xiaoyan Zhou[1] and Jianzhong Du[2,3*]

[1]State Key Laboratory of High-Efficiency Coal Utilization and Green Chemical Engineering, Ningxia University, Yinchuan 750021, China. [2]Department of Gynaecology and Obstetrics, Shanghai Fourth People's Hospital, School of Medicine, Tongji University, Shanghai 200434, China. [3]Department of Polymeric Materials, School of Materials Science and Engineering, Tongji University, 4800 Caoan Road, Shanghai 201804, China.

[*]correspondence to: Hui Sun (sunhui@nxu.edu.cn) and Jianzhong Du (jzdu@tongji.edu.cn)

This file includes:

Supplementary Text about characterizations and mathematical calculations

Supplementary Figures 1-27

1. Characterizations

1.1 Nuclear magnetic resonance (NMR)

The ^{1}H and ^{13}C NMR spectra of the monomer and PAzoMA were recorded using a Bruker AV 400 MHz spectrometer at room temperature with $CDCl_3$ as solvent.

1.2 High resolution mass spectroscopy (HRMS)

HRMS was performed on a Thermo Scientific Q Exactive Combined Quadrupole Orbitrap Mass Spectrometer by electrospray ionization and CH_2Cl_2 as solvent.

1.3 Gel permeation chromatography (GPC)

The molecular weight and polydispersity of PAzoMA were evaluated using a DMF GPC conducted by an Agilent 1260 Infinity GPC analysis system with two Shedex GPC KD series columns with HPLC grade DMF as the eluent at a flow rate of 0.8 mL min^{-1} at 40 ℃.

1.4 Differential scanning calorimetry (DSC)

DSC data was recorded using a DSC Q100 (TA Instruments). In the DSC measurement, the freshly extruded sample was kept at −80 ℃ for 3 min and heated at the rate of 10 ℃ min^{-1} to 150 ℃.

1.5 X-ray diffraction (XRD)

The crystalline structure of PAzoMA was characterized by powder XRD (Smart Lab) with a temperature control unit. The temperature was controlled from 25 to 110 ℃ with a heating rate of 10 ℃ min^{-1}.

1.6 Polarizing optical microscopy (POM)

The polarized micrograph photo of PAzoAA was obtained by an Olympus polarizing optical microscope (BX53P) equipped with a hot stage. The temperature was elevated to 110 ℃ with a heating

rate of 10 °C min^{-1}, and cooled to room temperature naturally. The photos were obtained at 110 and 25 °C, respectively.

1.7 Dynamic light scattering (DLS)

The hydrodynamic diameter and polydispersity of the nano-objects were determined by ZETASIZER Nano series instrument (Malvern Instruments ZS 90) at a fixed scattering angle of 90°. And the Zeta potentials of the nano-objects were also obtained.

1.8 UV-vis spectroscopy

The UV-vis spectra of the PAzoMA and the micelles were recorded by a UV759S UV–vis spectrophotometer (Shanghai Precision & Scientific Instrument Co., Ltd.). All the samples were analyzed using quartz cuvettes.

1.9 Transmission electron microscopy (TEM)

The aqueous solutions of the nano-objects were dropped onto the carbon film supported by copper grid and dried at ambient temperature. The images were recorded on a JEOL JEM-2100F instrument at 200 kV equipped with a Gatan 894 Ultrascan 1k CCD camera. SAED was conducted to analyze the crystal structure of the nano-objects.

2. Mathematical calculations

2.1 Calculation of the relationship between the diameter of the cylinders and the mass ratio of added spherical micelles to cylinders

Defining that: the diameter, length, amount and total volume of the preformed cylinders are d_0, l, y and V_0, respectively; the mass ratio of added spherical micelles to cylinders is n; the diameter and the total

volume of the grown cylinders are d_n and V_n, respectively, when the mass ratio of the added spherical micelles to cylinders is n. So the total volume of the preformed cylinders is

$$V_0 = \pi \frac{d_0^2}{4} ly \tag{1}$$

The total volume of the grown cylinders is

$$V_n = \pi \frac{d_n^2}{4} ly \tag{2}$$

Considering that

$$V_n = nV_0 + V_0 \tag{3}$$

So,

$$\pi \frac{d_n^2}{4} ly = (n + 1)\pi \frac{d_0^2}{4} ly \tag{4}$$

Equation (4) can be rewritten as

$$d_n^2 = (n + 1)d_0^2 \tag{5}$$

2.2 Calculation of the changes of the total specific surface area of spherical micelles before and after CD-FIPA

Defining that: the average radius of the spherical micelles is r_1; the amount of the micelles is x; the average radius of the cylinders is r_2; the average length of the cylinders is l; and the amount of the cylinders is y. We assume that the volume of the total spherical micelles doesn't change when transformed to cylinders. So,

$$\frac{4}{3}\pi r_1^3 x = \pi r_2^2 ly \tag{6}$$

The total specific surface areas of the spherical micelles (S_1) and cylinders (S_2) are obtained as Equations 6 and 7.

$$S_1 = 4\pi r_1^2 x \tag{7}$$

$$S_2 = \left(2\pi r_2^2 + 2\pi r_2 l\right) \tag{8}$$

So the ratio of the specific surface area of cylinders to that of spherical micelles is shown in Equation 9.

$$\frac{S_2}{S_1} = \frac{(2\pi r_2^2 + 2\pi r_2 l)y}{4\pi r_1^2 x} \tag{9}$$

It can be obtained from Equation 6 that

$$\frac{y}{x} = \frac{4r_1^3}{3r_2^2 l} \tag{10}$$

So Equation 4 could be rewritten as Equation 11.

$$\frac{S_2}{S_1} = \frac{4r_1}{3l} + \frac{2r_1}{3r_2} \tag{11}$$

Since $l >> r_1$, Equation 6 can be simplified as Equation 12.

$$\frac{S_2}{S_1} = \frac{2r_1}{3r_2} \tag{12}$$

2.3 Calculation of the changes of total specific surface area before and after the lateral growth of the cylinders

Defining that: the average radius of the spherical micelles is r_1; the amount of the micelles is x; the average radius of the cylinders is r_2 and r_3 before and after growth, respectively; the average length of the cylinders is l; and the amount of the cylinders is y. We assume that the volume of the total spherical micelles doesn't change when attached onto and fused with the cylinders. So,

$$\frac{4}{3}\pi r_1^3 x + \pi r_2^2 l y = \pi r_3^2 l y \tag{13}$$

The total specific surface areas of the added spherical micelles and the cylinders before and after growth are S_1, S_2 and S_3, respectively, as shown in Equations 14-16.

$$S_1 = 4\pi r_1^2 x \tag{14}$$

$$S_2 = \left(2\pi r_2^2 + 2\pi r_2 l\right) \tag{15}$$

$$S_3 = \left(2\pi r_3^2 + 2\pi r_3 l\right) \tag{16}$$

So the ratio of the specific surface area of the grown cylinders to that of spherical micelles and that of immature cylinders is shown in Equation 17.

$$\frac{S_3}{S_1 + S_2} = \frac{(2\pi r_3^2 + 2\pi r_3 l)y}{4\pi r_1^2 x + (2\pi r_3^2 + 2\pi r_3 l)y} \tag{17}$$

It can be obtained from Equation 13 that

$$x = \frac{3(r_3^2 - r_2^2)ly}{4r_1^3} \tag{18}$$

So Equation 17 can be rewritten as

$$\frac{S_3}{S_1 + S_2} = \frac{2r_1 r_3 y(r_3 + l)}{3\left(r_3^2 - r_2^2\right)ly + r_2 y(r_2 + l)} \tag{19}$$

Considering that $l >> r_2$, r_3, Equation 19 can be simplified as

$$\frac{S_3}{S_1 + S_2} = \frac{2r_1 r_3}{3\left(r_3^2 - r_2^2\right) + r_2} \tag{20}$$

3. Supplementary Figures

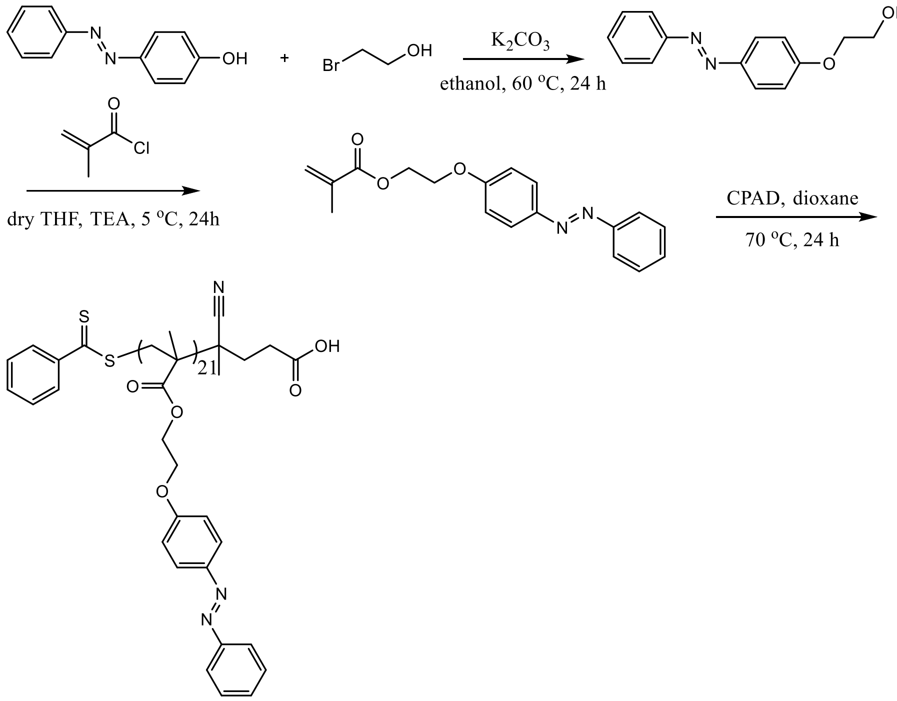

Supplementary Fig. 1 Synthetic route to PAzoMA homopolymer.

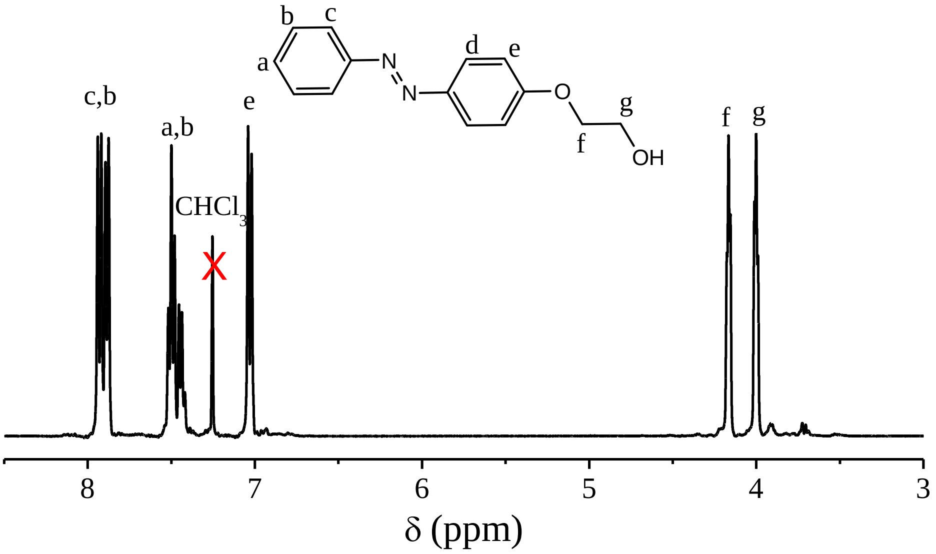

Supplementary Fig. 2 ^{1}H NMR spectrum of 2-(4-(phenyldiazenyl)phenoxy)ethanol.

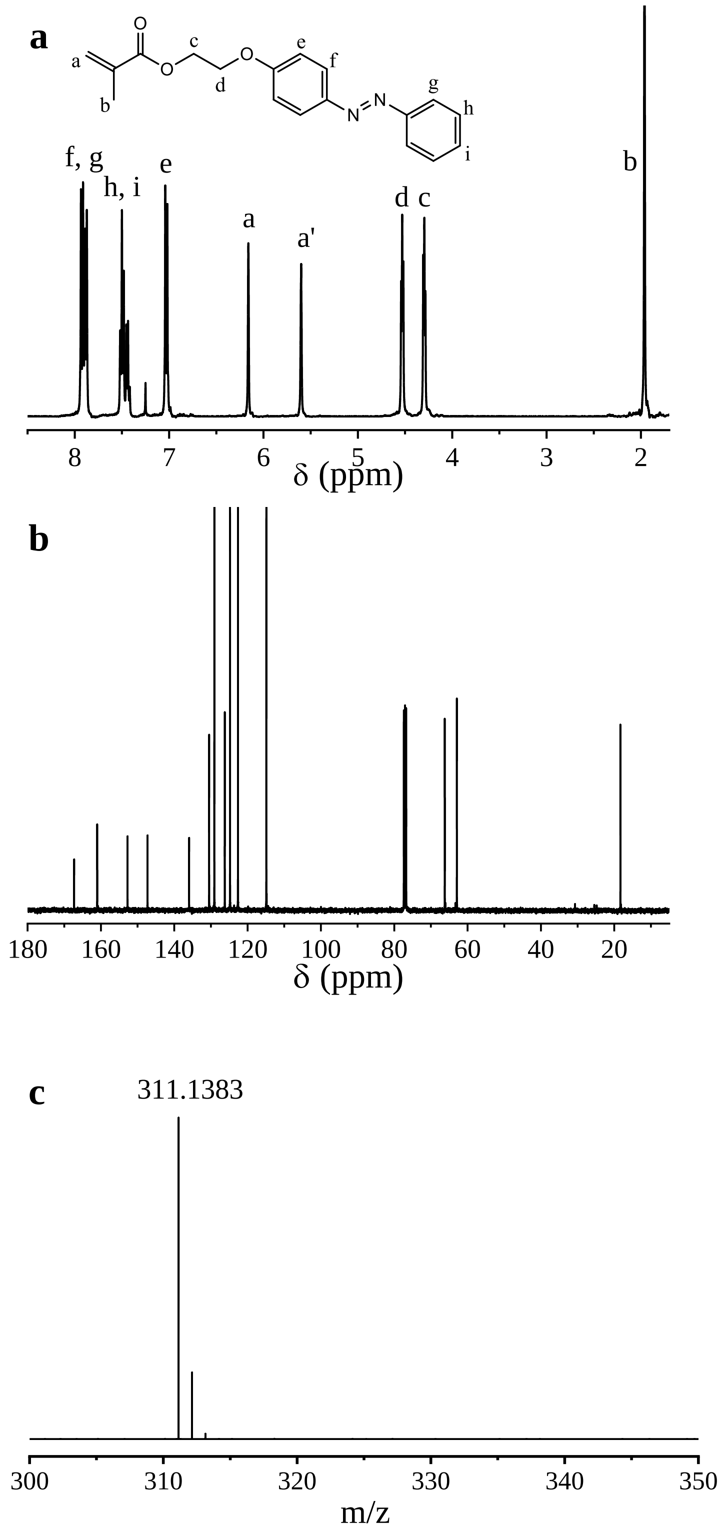

Supplementary Fig. 3 NMR spectra and HRMS of AzoMA monomer. a ^{1}H and **b** ^{13}C NMR spectra of AzoMA monomer in $CDCl_3$. **c** ESI mass spectrum of AzoMA monomer.

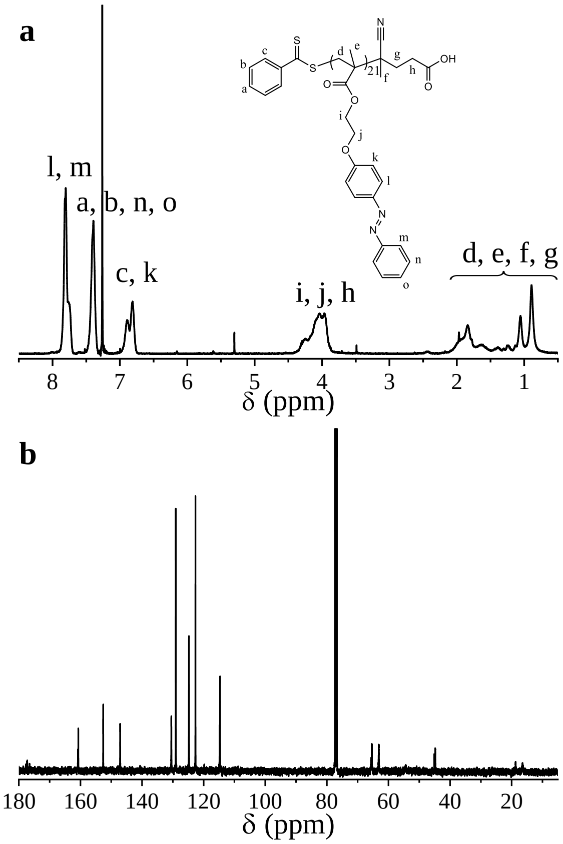

Supplementary Fig. 4 NMR spectra of PAzoMA homopolymer. a ^{1}H and **b** ^{13}C NMR spectra.

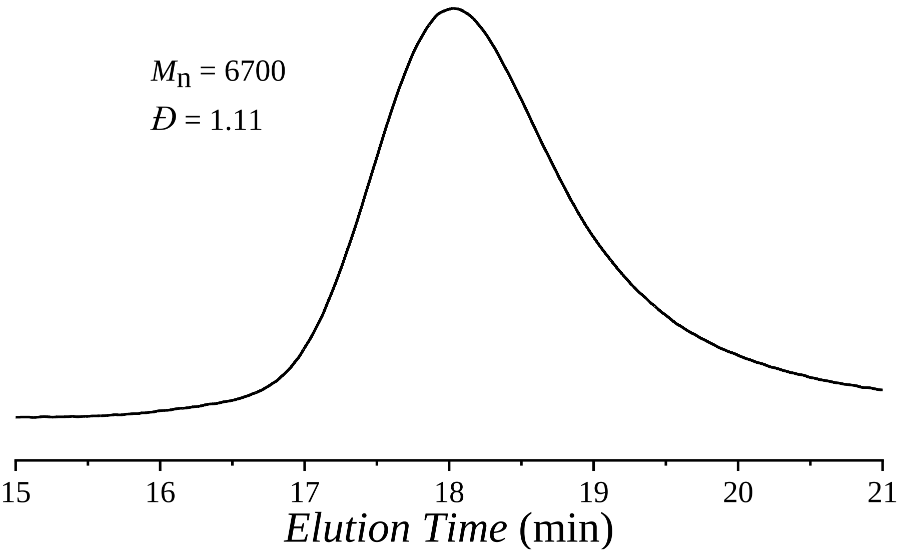

Supplementary Fig. 5 GPC trace of PAzoMA homopolymer.

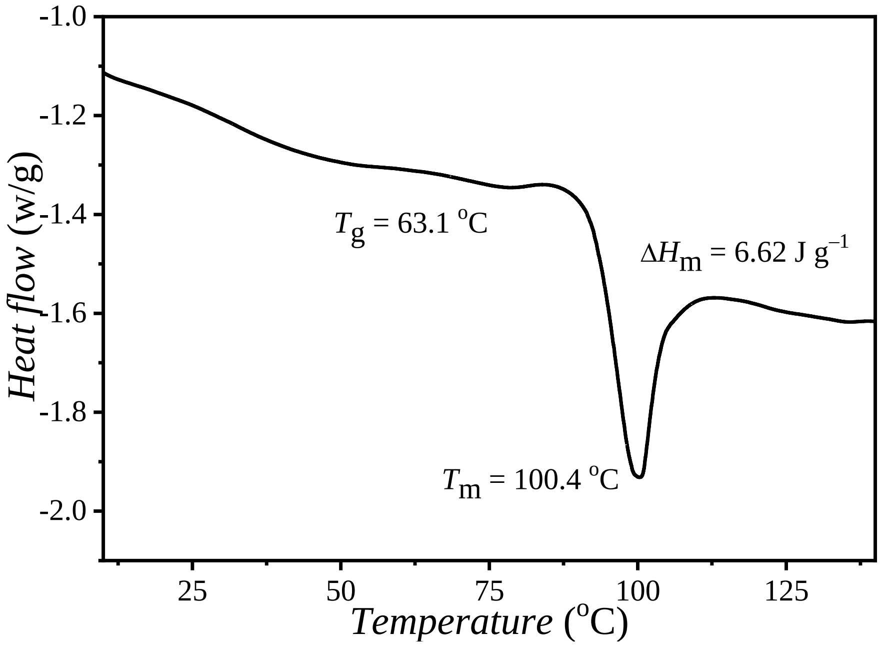

Supplementary Fig. 6 DSC isotherm of PAzoMA homopolymer.

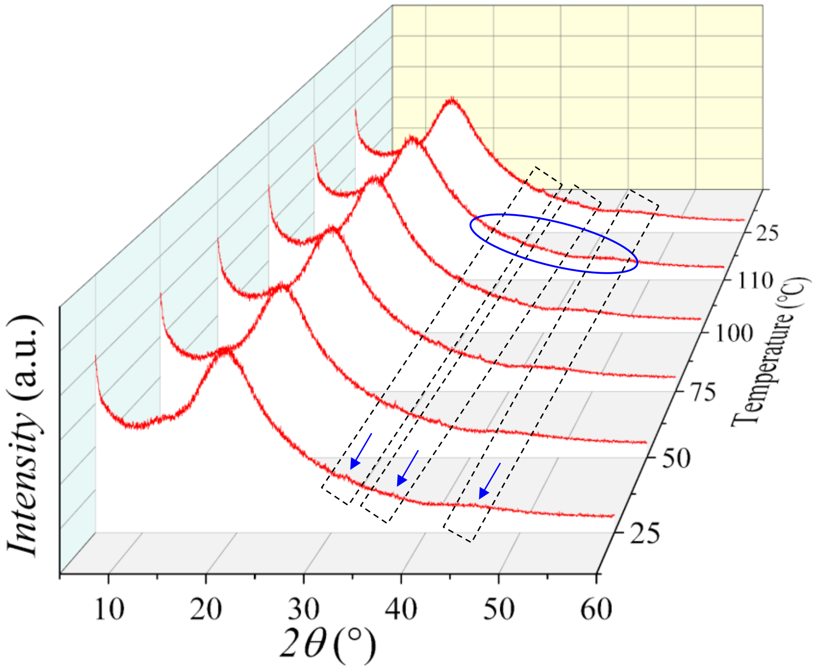

Supplementary Fig. 7 XRD patterns of PAzoMA homopolymer at different temperatures.

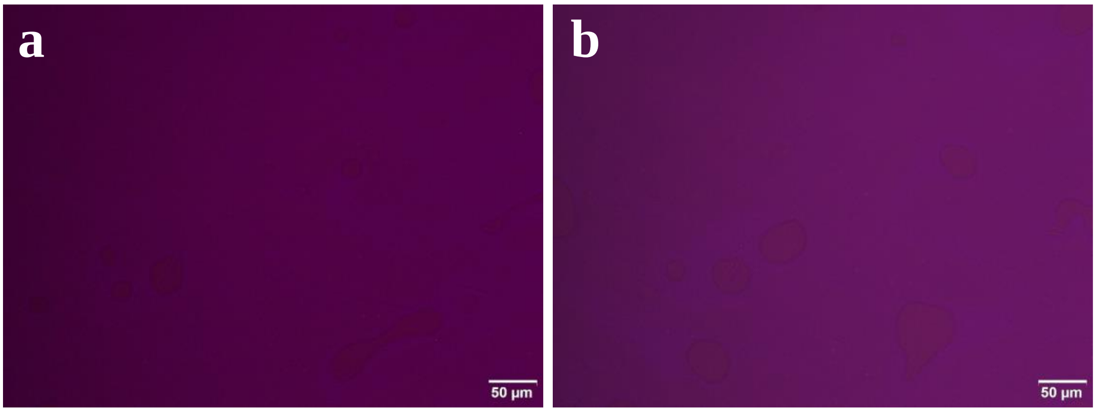

Supplementary Fig. 8 POM images of PAzoMA homopolymer at different temperatures. a 110 ℃ and **b** 25 ℃.

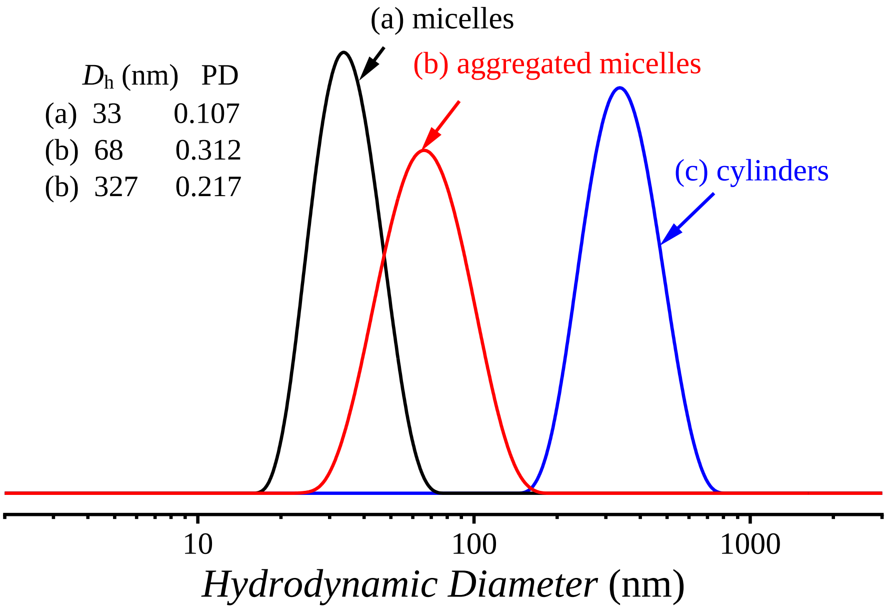

Supplementary Fig. 9 DLS results of spherical micelles, aggregated micelles and cylinders.

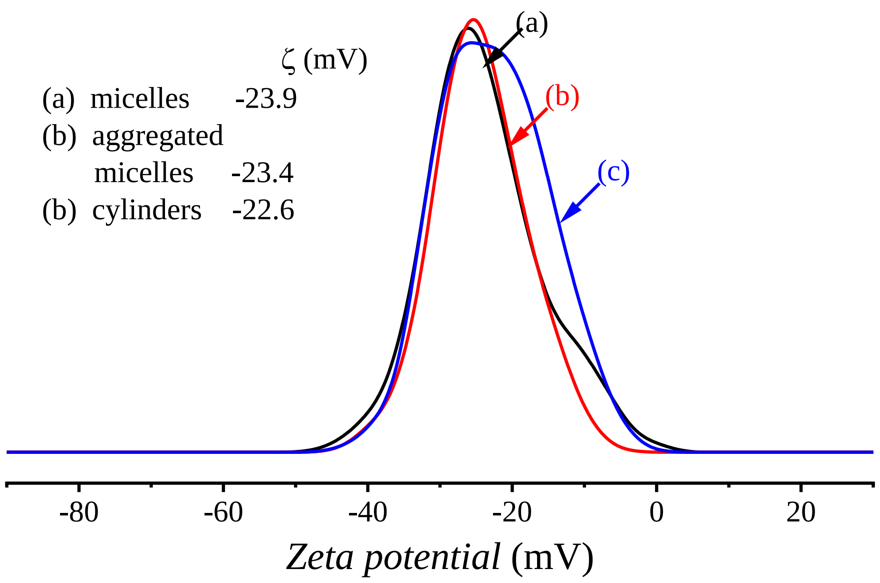

Supplementary Fig. 10 Zeta potentials of spherical micelles, aggregated micelles and cylinders.

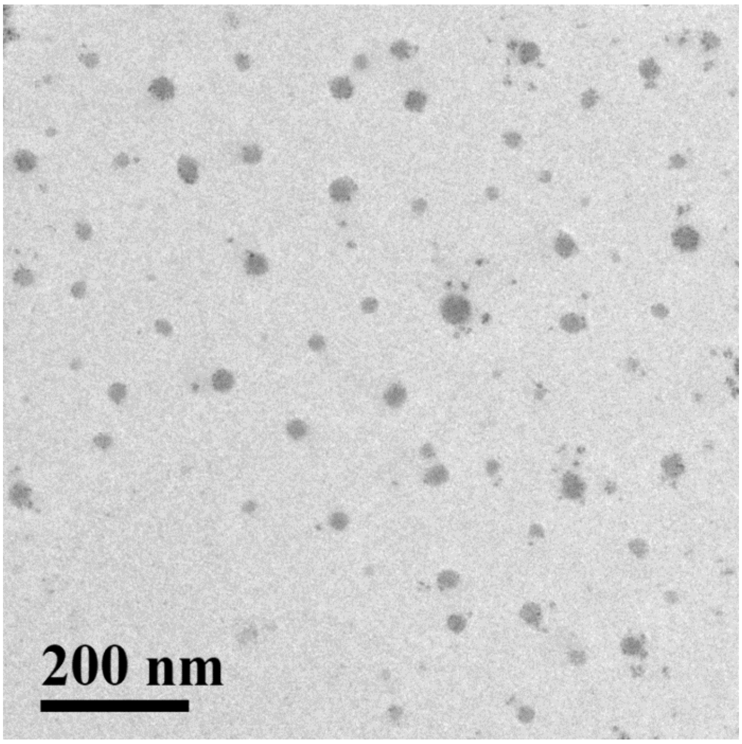

Supplementary Fig. 11 TEM image of spherical micelles before incubation.

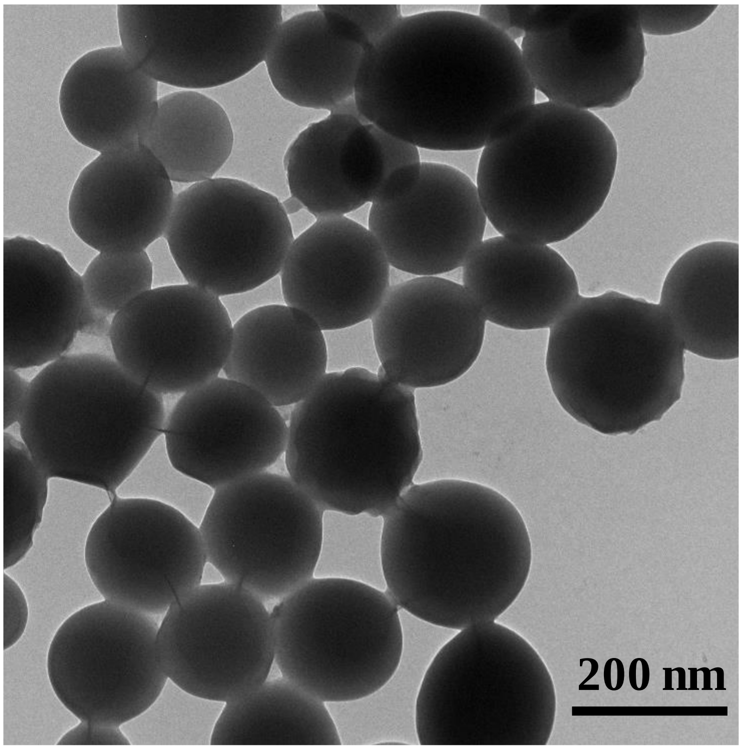

Supplementary Fig. 12 TEM image of large compound micelles when decreasing the water dropping rate to 0.1 mL min^{-1}.

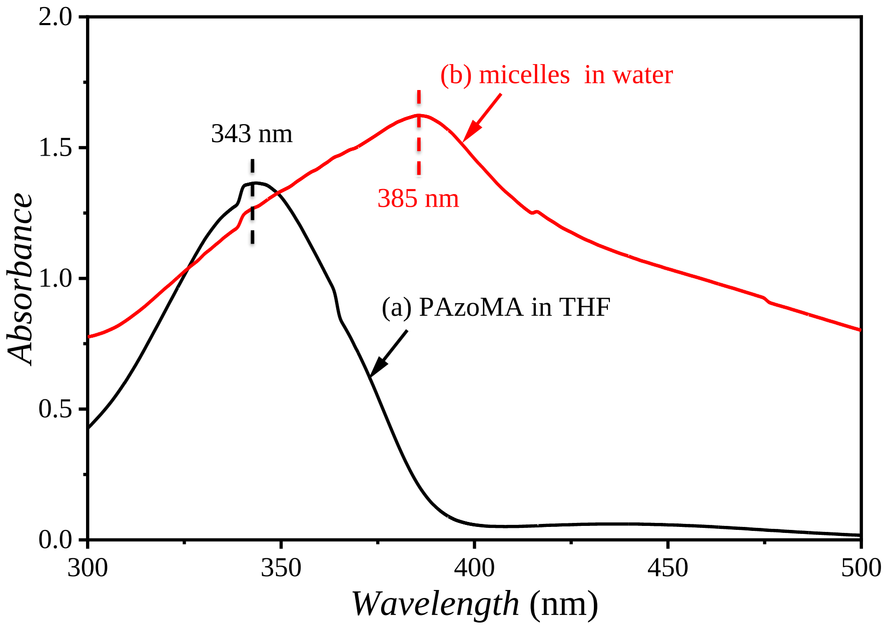

Supplementary Fig. 13 UV-vis spectra of (a) PAzoMA homopolymer in THF and (b) spherical micelles in water.

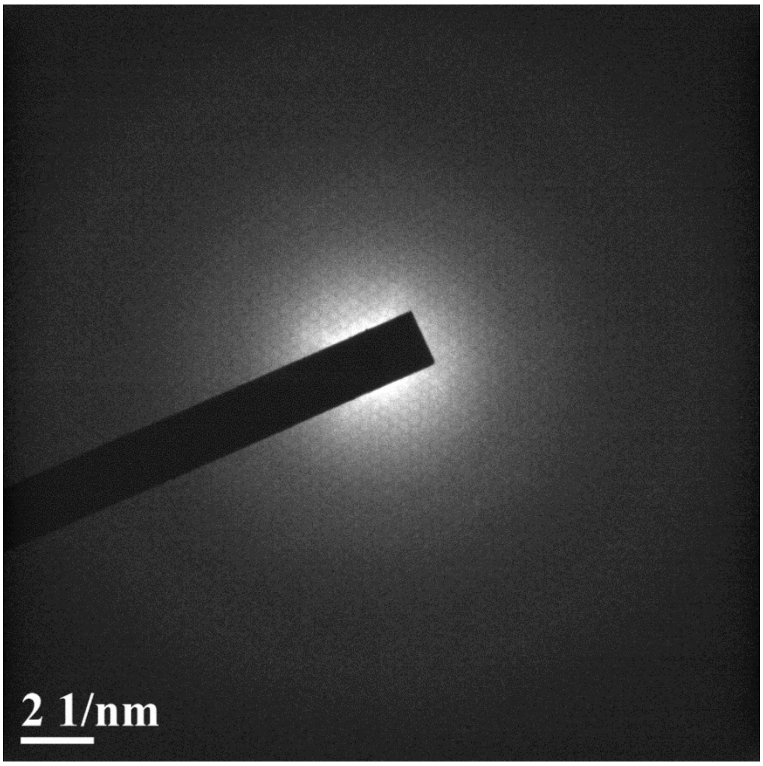

Supplementary Fig. 14 The SAED of spherical micelles before incubation.

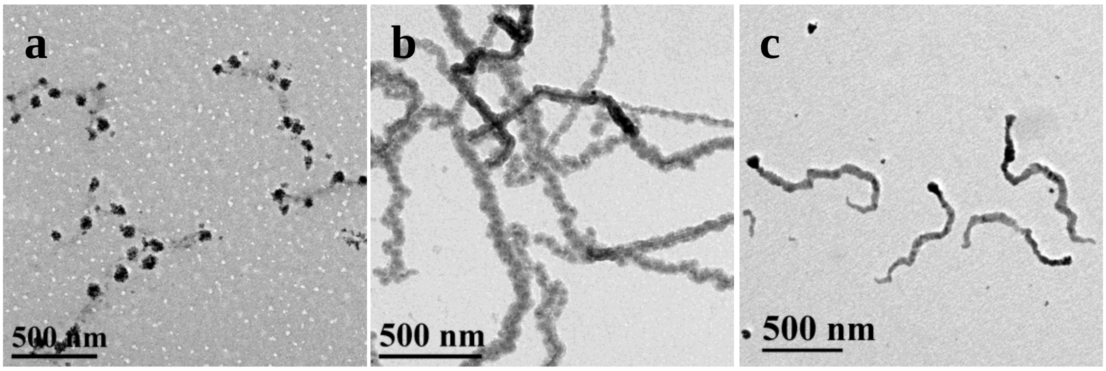

Supplementary Fig. 15 Formation of cylinders *via* the CD-FIPA of spherical micelles at a concentration of 0.25 mg mL^{-1}. a Incubated for 30 min, **b** 90 min and **c** 6 h.

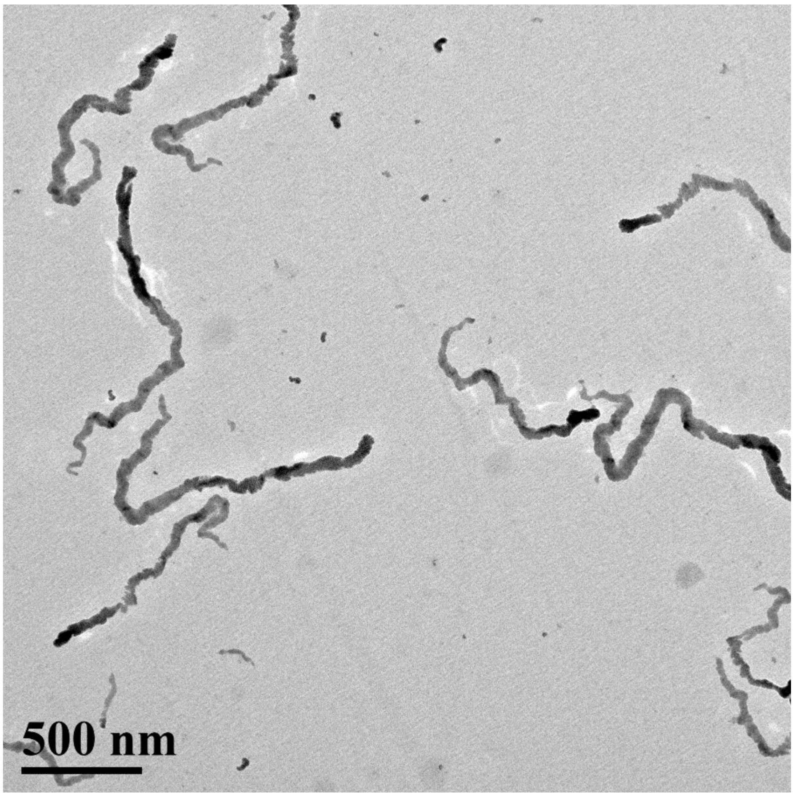

Supplementary Fig. 16 Low-magnification TEM image of cylinders by CD-FIPA.

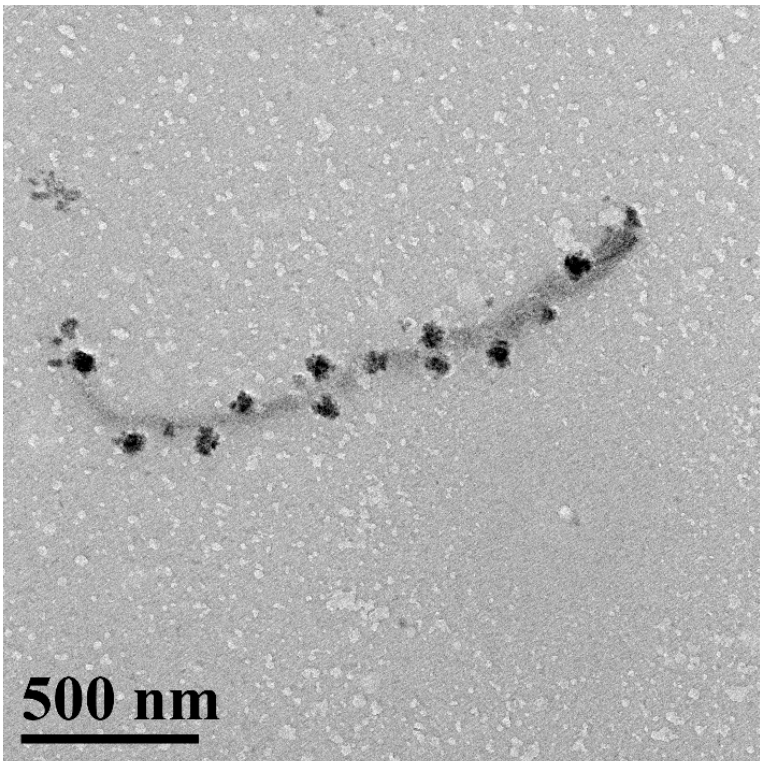

Supplementary Fig. 17 TEM image showing the origin of cylinders with a thick head and thin tail.

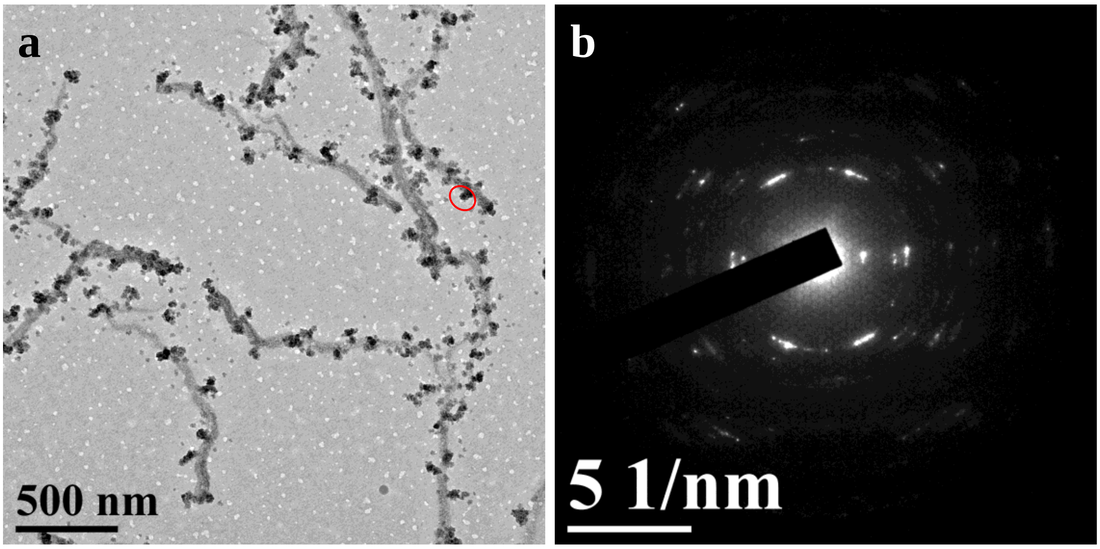

Supplementary Fig. 18 SAED pattern of lateral grown section of cylinders. a TEM image and **b** corresponding SAED pattern of the red circle in **a**.

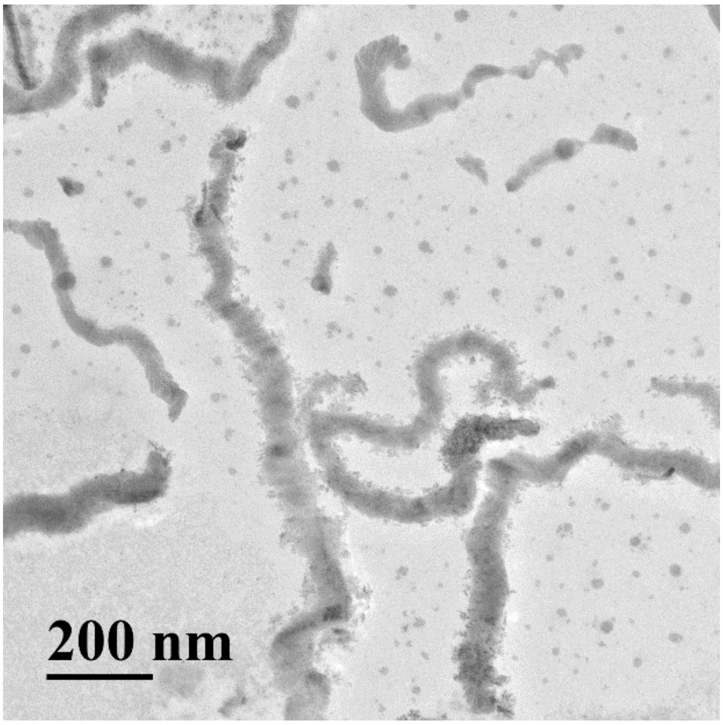

Supplementary Fig. 19 TEM image of the mixed solution of spherical micelles and cylinders kept at 25 °C for 3 h.

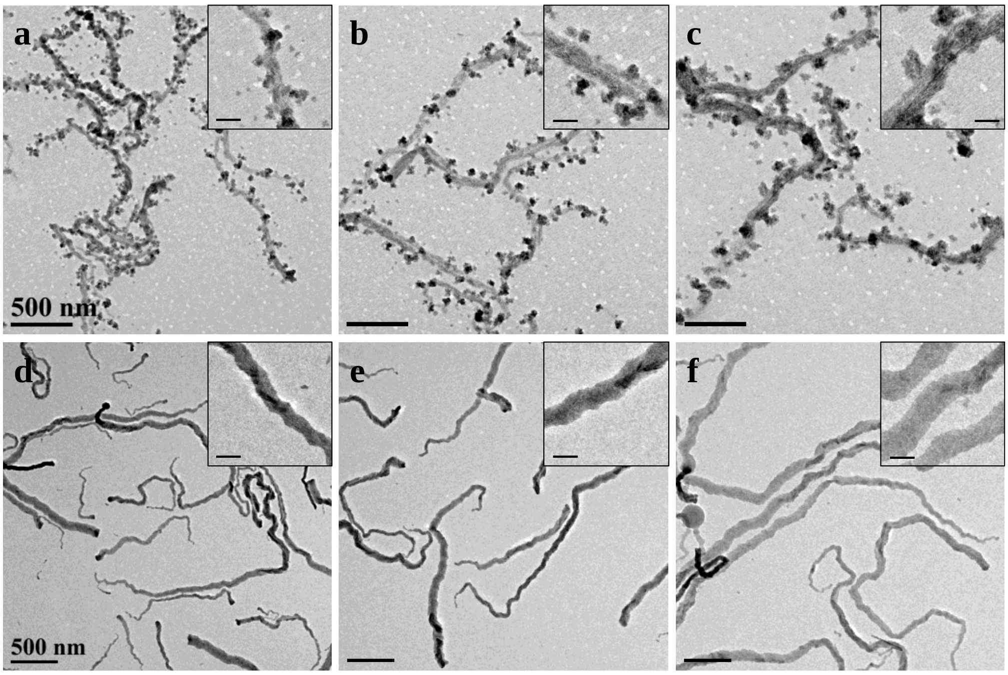

Supplementary Fig. 20 Supplamentary TEM images of living lateral growth of cylinders. a-c incubation of cylinders and added micelles at 75 °C for 60 min; **d-f** for 3 h. **a, d** first growth, **b, e** second growth, **c, f** third growth. The scale bars of **a-f** and the insets are 500 and 100 nm, respectively.

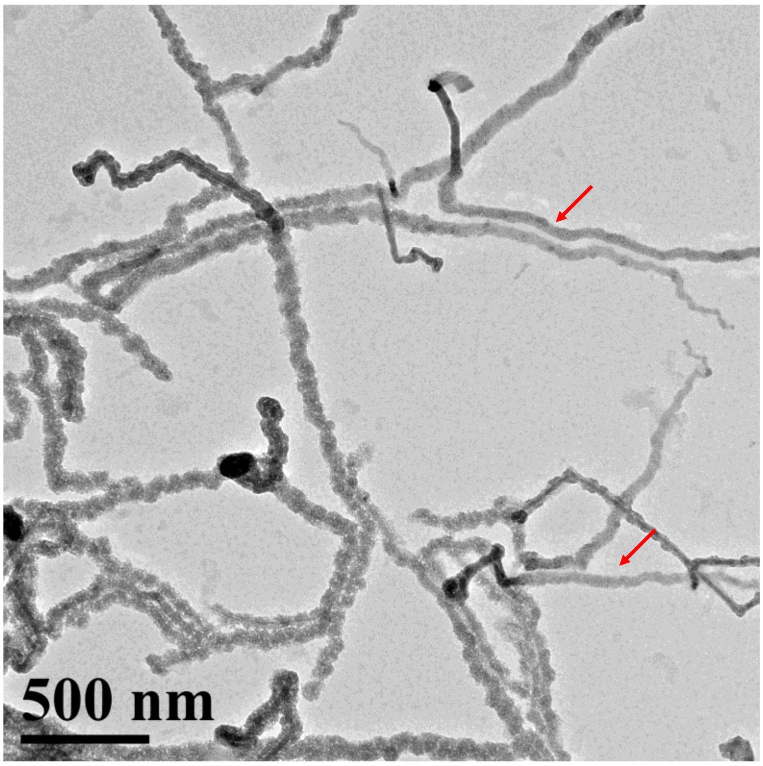

Supplementary Fig. 21 TEM image of growing cylinders.

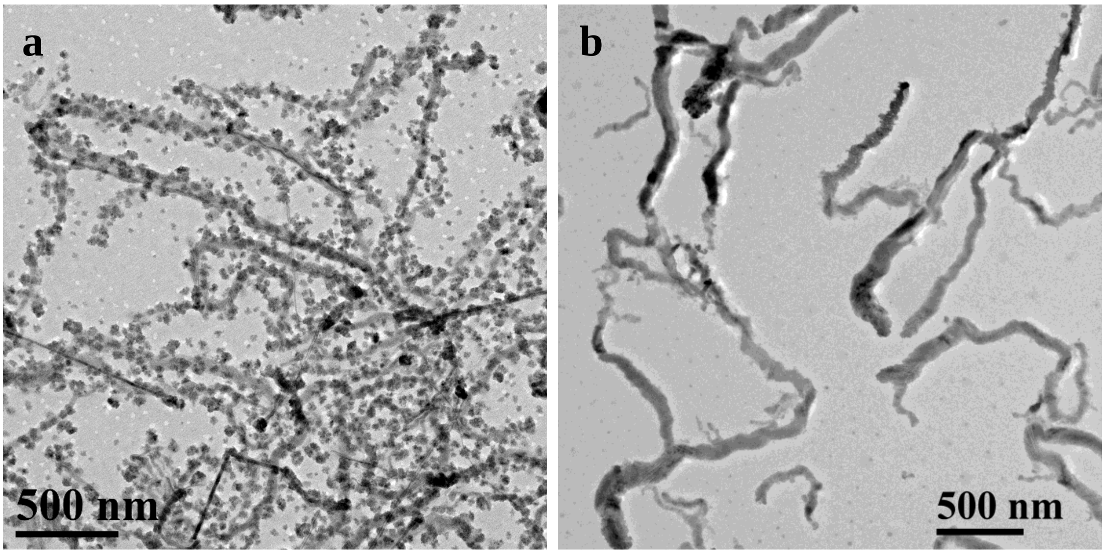

Supplementary Fig. 22 TEM images of the living lateral growth cylinders when increasing the amount of added spherical micelles to two times of that before. a incubation of cylinders and added micelles at 75 °C for 60 min and **b** for 3 h.

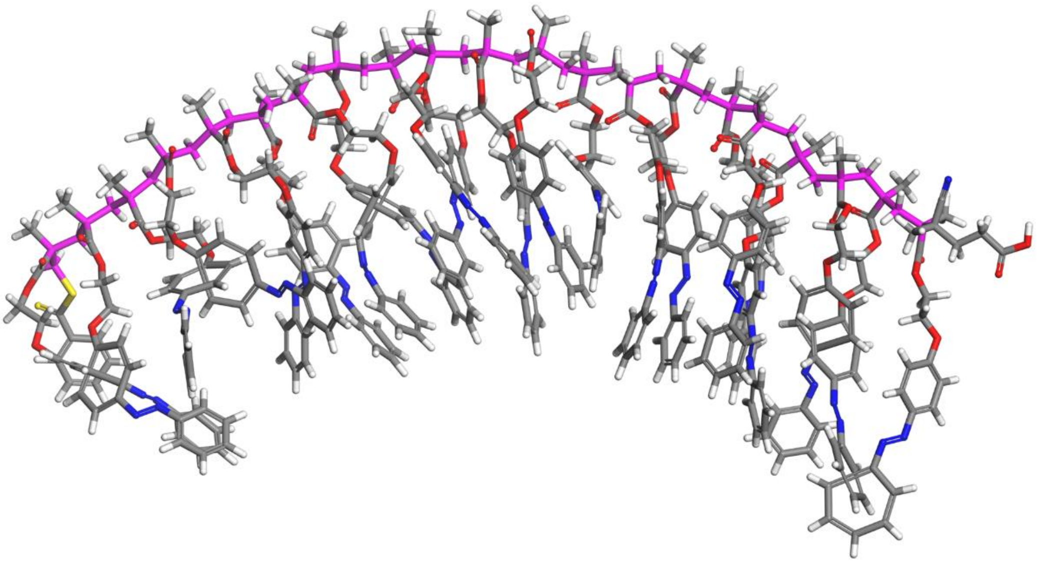

Supplementary Fig. 23 Forcite-optimized model of PAzoMA$_{21}$. The purple chain represents the backbone of the polymer. Different colors of atoms represent different elements: grey for carbon, white for hydrogen, blue for nitrogen, red for oxygen and yellow for sulfur.

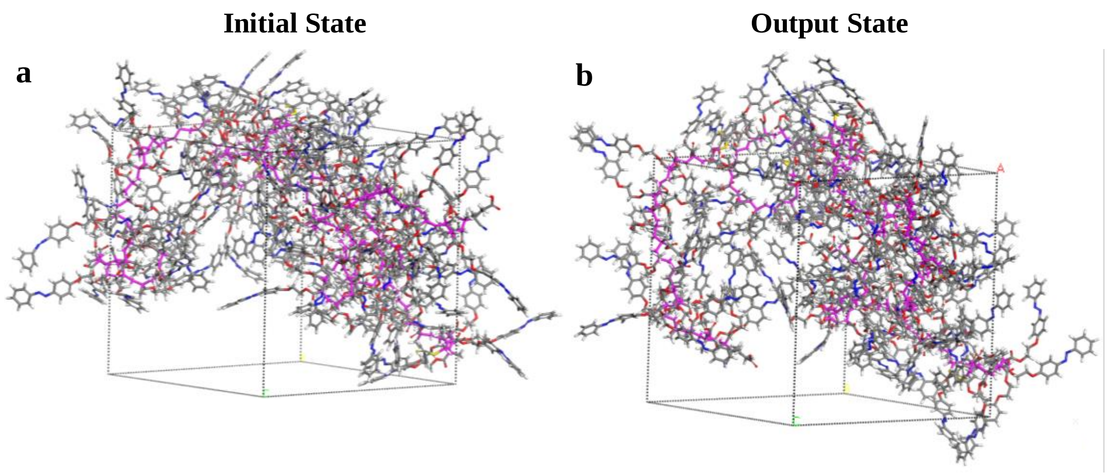

Supplementary Fig. 24 Molecular geometry of PAzoMA$_{21}$. a Initial state and **b** Output state.

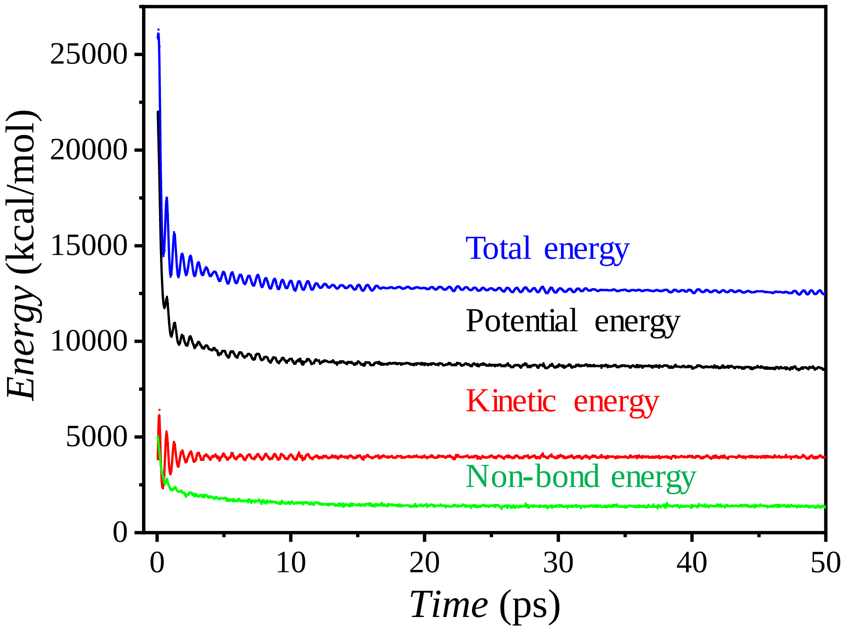

Supplementary Fig. 25 Energy evolution of the cell during the simulation process.

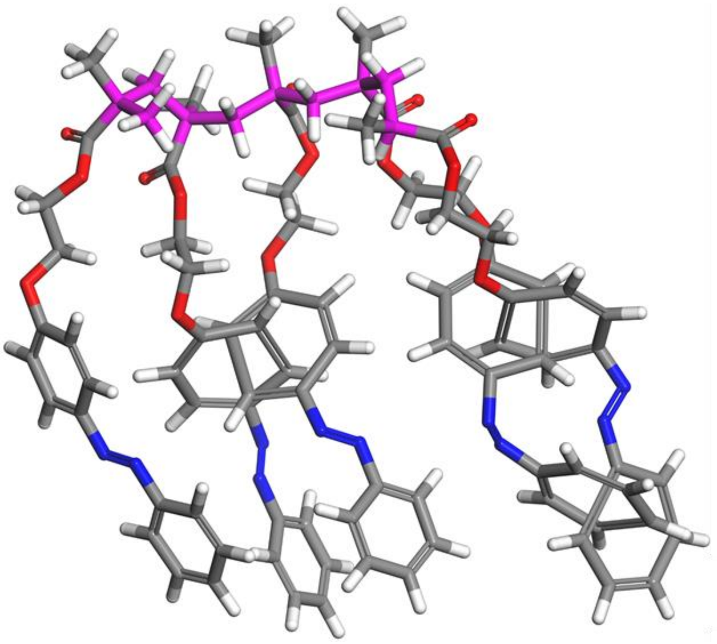

Supplementary Fig. 26 Forcite-optimized model of $PAzoMA_5$. The purple chain represents the backbone of the polymer. Different colors of atoms represent different elements: grey for carbon, white for hydrogen, blue for nitrogen, red for oxygen and yellow for sulfur. To simplify the calculation process, $PAzoMA_5$ without terminal group was used to reveal the energy evolution during CD-FIPA.

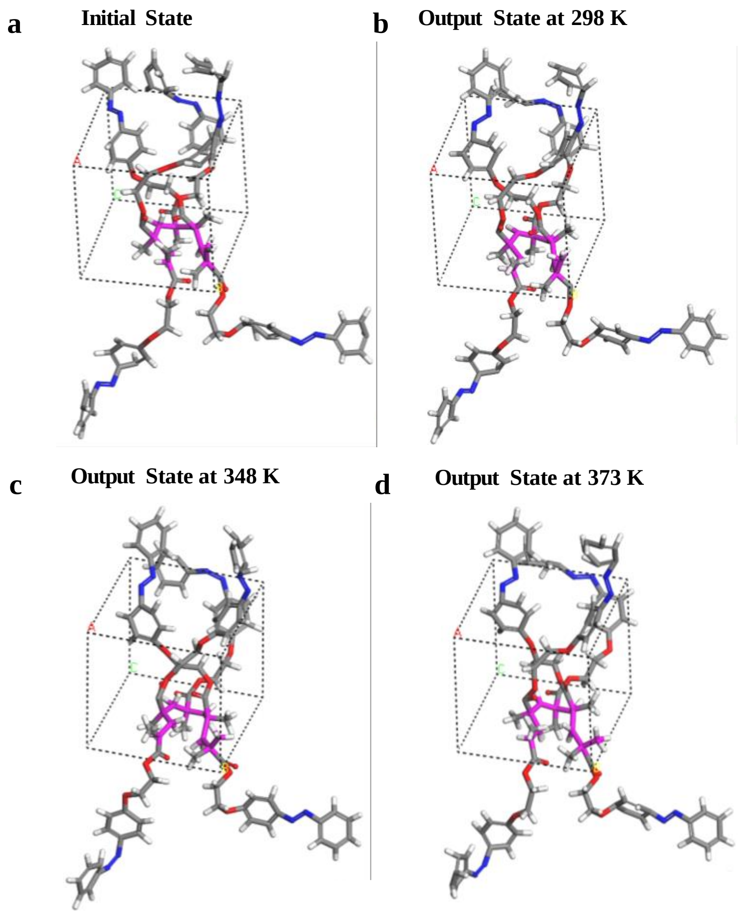

Supplementary Fig. 27 Molecular geometry of PAzoMA$_5$ at different temperatures. a Initial state, **b** 298 K, **c** 348 K and **d** 373 K.
